# *Helicobacter canis*: A Review of Microbiological and Clinical Features

**DOI:** 10.3389/fmicb.2021.814944

**Published:** 2022-02-23

**Authors:** Benjamin Lardinois, Leïla Belkhir, Alexia Verroken

**Affiliations:** ^1^Department of Microbiology, Cliniques universitaires Saint-Luc, Université catholique de Louvain, Brussels, Belgium; ^2^Department of Internal Medicine and Infectious Diseases, Cliniques universitaires Saint-Luc, Université catholique de Louvain, Brussels, Belgium

**Keywords:** *Helicobacter canis*, bacteriology, bacteremia, susceptibility testing, mini-review, clinical features

## Abstract

*Helicobacter canis*, an enterohepatic Helicobacter, has proven its role in human diseases and has been rediscussed in recent years as its zoonotic potential is increasingly described. Routine microbiological detection of this pathogen is a difficult task as its culture may fail due to fastidious growth. It is therefore supposed that many clinical laboratories under-recognize *H. canis* infections. A review of all clinical and microbiological literature currently available from previous relevant *H. canis* human clinical cases, mainly bacteremia, added with a clinical case observed at the Cliniques universitaires Saint-Luc, was performed. Clinical features of *H. canis* reports show the presence of underlying clinical conditions in 89% of the cases, bacteremia in 83%, associated fever in 58%, and recent close contact with pets in 83%, especially dogs. The observed microbiological trends from 10 cases of bacteremia were a median of 4 days until positive blood culture bottle detection, subcultures showing a thin layer of small colonies under microaerophilic atmosphere at 35–42°C after 3–4 days of growth, and an identification requiring 16S rRNA sequencing given the difficulties observed with MALDI-TOF MS. Low MICs were observed for penicillins, amoxicillin/clavulanic acid, carbapenems, and metronidazole in opposition to high MICs for ciprofloxacin. A frequent association of *H. canis* and bacteremia in immunocompromised patients with recurrent fever in contact with pets, especially dogs, was identified. Considering the fastidious growing capacities, final identification from blood cultures may not be expected before 7 days. Intravenous ceftriaxone, oral doxycycline, or metronidazole has been suggested as efficient therapeutic choices.

## Introduction

Initially discovered by Stanley et al. (1993) from the feces of dogs, *Helicobacter canis* has been rediscussed in recent years as its zoonotic potential is increasingly described. Several *Helicobacter* species find their reservoirs in animals even for *H. pylori* (Momtaz et al., 2014). Non-pylori *Helicobacter* species and especially enterohepatic *Helicobacter* including *H. canis* have proven their role in human diseases (Ménard et al., 2014; Liu et al., 2015). Swennes et al. (2014) identified sheep as a potential reservoir, and Sabry et al. (2016) reported an indisputable zoonotic transmission by comparing *H. canis* sequences from humans and sheep contacts. Interestingly, nine clinical cases with *H. canis* bacteremia including the current one reported close contacts with cats or dogs (Gerrard et al., 2001; Leemann et al., 2006; Prag et al., 2007; Alon et al., 2010; Abidi et al., 2013; van der Vusse et al., 2014; Shakir et al., 2017; Mihevc et al., 2021). Seven of them, including ours, concerned patients with underlying conditions (Gerrard et al., 2001; Alon et al., 2010; Abidi et al., 2013; van der Vusse et al., 2014; Shakir et al., 2017; Mihevc et al., 2021).

Detection of *H. canis* by a microbiology laboratory is a difficult task under routine conditions as culturing of *Helicobacter* species may fail due to fastidious growth. Therefore, many clinical laboratories may under-recognize this germ, and the prevalence of this organism might hereby be underestimated. Microbiologists as well as clinicians should keep in mind its potential for clinical involvement. Only laboratories with gene sequencing capability are able to reliably identify the pathogen from clinical specimens. An optimal communication between microbiologists and physicians is therefore crucial.

To our knowledge, three cases have investigated the antimicrobial susceptibility of *H. canis* strains (Leemann et al., 2006; Prag et al., 2007; Mihevc et al., 2021). Here we described a case of *H. canis* bacteremia in an immunocompromised woman suffering from end-stage renal disease with a possible zoonotic transmission. We also summarized data from previous relevant *H. canis* human clinical cases reviewing the pathogen, its susceptibility profile, and the frame of infectious diseases caused.

## Case Study

A 55-year-old woman initially consulted a nephrologist for renal transplantation follow-up. She had undergone a renal transplant 13 years earlier after being diagnosed with an autosomal dominant polycystic kidney disease but the graft had become gradually dysfunctional. Additional comorbidities included secondary hyperparathyroiditis and moderate anemia as well as a long-standing splenectomy. At the time of consultation, she described flu-like symptoms and fatigue in addition to increasing diarrhea. The dosage of mycophenolate mofetil (MMF), part of her immunosuppressive therapy, was therefore temporarily reduced. Interestingly, she had presented several episodes of shivers few days earlier but had not measured body temperature at home. Clinical examination revealed a traumatic pretibial wound with an associated slight edema. However, no cellulitis was observed despite a fragile skin under cortisone conditions.

Additional testing included blood culture sampling composed of two sets of a BACTEC Plus Aerobic/F culture vial and a BACTEC Plus Anaerobic/F culture vial (Becton Dickinson Diagnostic Systems, Sparks, MD, United States) and incubated in a Bactec 9240 instrument (Becton Dickinson). Bacterial growth was detected in a single aerobic bottle 3 days and 4 h following incubation. Gram revealed Gram-negative, spiral-shaped micro-organisms. No acridine orange stain was performed. The positive bottle was subcultured on Columbia 5% sheep blood agar in a 5% CO_2_ atmosphere at 35°C according to routine laboratory workflow. As no growth was observed, additional subcultures were initiated on a horse blood enriched with co-factor V and X agar, a Gram-negative selective and differentiating medium, a 5% horse blood Brucella agar, and a Campylobacter medium supplemented with activated charcoal, all incubated at 35°C in a 7.2% CO_2_ and 6% O_2_ atmosphere (equals microaerophilic conditions). These subsequent subcultures all successfully recovered colonies within 3–5 days. Optimal growth was obtained on Brucella 5% sheep blood agar in microaerophilic conditions showing a thin layer of colonies with a cloud-like appearance after 3 days (see Supplementary Figure 1). The micro-organism was oxydase positive. Unfortunately, no identification was possible using matrix-assisted laser desorption ionization-time of flight mass spectrometry (MALDI-TOF MS) (Microflex LT; Bruker Daltonics, Bremen, Germany).

Ultimately, identification was realized by partial sequencing of 1458 base pairs of the 16S ribosomal ribonucleic acid (rRNA) gene of which 1,213 base pairs were analyzable on a Genetic Analyzer ABI 3730XL (Applied Biosystems; Invitrogen Life Technologies, Carlsbad, CA, United States), with the BigDye Terminator kit (Applied Biosystems) using a laboratory-developed method (Wauters et al., 2003). It revealed 99.9% identity correspondence representing *Helicobacter canis* 16S rRNA gene sequences after Basic Local Alignment Search Tool (BLAST) analysis of the consensus. The nucleotide sequence was submitted to GenBank and obtained the accession number AY631946.

Antimicrobial susceptibility testing (AST) using *E*-tests (bioMérieux, Marcy-l’Etoile, France) was performed for minimum inhibitory concentration (MIC) determination. The *E*-tests were placed on three McFarland inoculum Brucella agar plates and incubated in microaerophilic conditions for 72 h according to the manufacturer’s recommendations (see Supplementary Figure 2). Microbiological investigations and MICs reported to the physicians are summarized in Supplementary Table 1. *H. pylori*-related EUCAST 2021 breakpoints were used for rifampicin, metronidazole, and tetracycline since there were no species-specific breakpoints or other recommendations for these antibiotics. PK-PD EUCAST 2021 breakpoints were used for the other antibiotics (The European Committee on Antimicrobial Susceptibility Testing, 2021). No breakpoints were considered for clindamycin MIC.

Interestingly, a slight increase of C-reactive protein the day of venipuncture was noticed compared to a previous blood test 2 weeks earlier. The white blood cells were within the reference range (RR) (8.8 × 10^3^ cells/μl; RR 4.0–10.0 × 10^3^ cells/μl). Total IgG were slightly below reference range (6.8 g/L; RR 7.0–16.0 g/L). Other blood test results realized during follow-up showed no other acute abnormalities. Urine analysis reported a slight increase of white blood cells (8 cells/field; RR < 5 cells/field) and moderate bacteriuria. Urine culture showed a mixed contamination flora and stool cultures did not grow with any enteropathogenic germ despite the use of a *Campylobacter* medium supplemented with activated charcoal and a blood-based *Campylobacter* medium.

The patient was admitted to the hospital 20 days after her follow-up appointment in order to treat the bacteremia. Four sets of blood cultures were sampled with an extended 14-day incubation time, yet all were reported negative. A swab from the persistent tibial wound was cultured according to identical conditions as described for the blood culture bottles, but *H. canis* remained undetected. Similarly, we failed to detect the strain in the patient’s stool. A bacteriological culture was also realized on the patient’s dog’s stool; however, it did not reveal the germ. Additional urine and stool cultures showed results with no clinical relevance during the 2 weeks of hospitalization.

The patient received an intravenous ceftriaxone 2 g/day antibiotic therapy for 6 weeks.

She was eventually cured and continued to be monitored every 2 weeks for her chronic kidney disease.

## Discussion

This review aimed at identifying the common features of previous and current *H. canis* bacteremia to assist clinicians in the management of these patients. Two case reports without bacteremia are also discussed. To the best of our knowledge, 12 human clinical cases including the current one have already been reported about *H. canis*, but recommendations are scarce. Initially discovered in 1993 from the feces of healthy or diarrheic dogs (Stanley et al., 1993), then clinically reported the same year in a boy with gastroenteritis (Burnens et al., 1993), this enterohepatic pathogen has proven its involvement over the recent years in bacteremia in patients with and without co-morbidities.

Surprisingly, the typical reservoir of this species is not fully understood as no epidemiologic studies have been performed (van der Vusse et al., 2014). However, several studies identified *H. canis* as a dominant *Helicobacter* in the digestive system of both healthy and unhealthy dogs and cats (Stanley et al., 1993; Fox et al., 1996; Foley et al., 1999; Shen et al., 2001; Ekman et al., 2013; Tabrizi and Atashi, 2015; Ochoa et al., 2021b). Unlike other enterohepatic *Helicobacter* species, *H. canis* may induce an active host immune response as observed in an experimental model using the wax moth larvae *Galleria mellonella* (Ochoa et al., 2021a), supporting previous observations of infection in immunocompromised hosts. Recently, the bacteria was identified in sheep feces (Swennes et al., 2014; Sabry et al., 2016). One study proved zoonotic transmission of the germ by obtaining identical sequences of identity matrix in sheep and its animal caretaker. The contamination would have occurred by milk, animal carcass, or potentially through direct fecal-oral route (Sabry et al., 2016). Transmission of commensal *Helicobacter* species living in the intestines of animals is not obvious although the latter route is the most likely (Ekman et al., 2013; van der Vusse et al., 2014). Lack of urease activity, bile tolerance, and fecal source could explain a preferential digestive carriage in the lower intestinal tract (Stanley et al., 1993; Alon et al., 2010). Although possible carriage of *H. canis* in humans needs further investigations, it could explain translocation from the intestines to the bloodstream once the mucosa is damaged, either by a virus (Burnens et al., 1993) or by an immunosuppressive therapy such as MMF as cited in this report and in a previous one (Abidi et al., 2013). Interestingly, close contacts with pets mainly with dogs was reported in 83% of clinical cases including this one reinforcing the hypothesis of zoonotic transmission by a companion animal. The source of the bacteremia could not be confirmed in many cases since patients experienced skin abnormalities and/or digestive symptoms with underlying conditions that possibly contributed to the entry of the germ in the bloodstream. Our clinical case reflected this ambiguity as the immunocompromised patient had a skin wound and increasing diarrhea.

Previous and current microbiological investigations are summarized in Table 1. Although anaerobic blood bottles were reported positive in two cases (Alon et al., 2010; van der Vusse et al., 2014), aerobic blood bottles were more likely to detect *H. canis* which is in accordance with the microaerophilic atmosphere required for the germ’s growth. Pediatric blood bottles enriched with yeast or meat extract with less sodium polyanetholsulfonate were also described to be suitable for the pathogen’s detection (Prag et al., 2007; Gutiérrez-Arroyo et al., 2017). Interestingly, about half of the aerobic blood bottles sampled on patients’ admissions were reported positive with an average time-to-detection of 4 days. This delay is within the classical 5-day blood culture incubation period applied by most clinical microbiology laboratories. Acridine orange and Gram staining described both curved- and spiral-shaped rods. Two case-reports related the fact that enterohepatic *Helicobacter* are difficult or even impossible to subculture (Alon et al., 2010; van der Vusse et al., 2014). The use of enriched non-selective media such as 5% sheep blood, chocolate, or Brucella agar which are commonly used in bacteriology laboratories appeared to be appropriate. On the other hand Mueller–Hinton media did not allow the growth of *H. canis* (Ochoa et al., 2019). Optimal growth was obtained under microaerophilic atmosphere at 35–42°C and the suspicious bacterial colonies were visible to the naked eye after an average of 3–4 day-incubation. Identification of *H. canis* was particularly difficult by standard methods considering its weak biochemical reactivity. Even MALDI-TOF MS was not suitable given the high number of identification failures in previous reports. Only two reports partially succeeded in identifying *H. canis* by this technique with a score of 1.93 and 1.88, respectively (Abidi et al., 2013; Mihevc et al., 2021). However, no reliable identification at genus level (log score ≥1.7) or species level (log score ≥2.0) is possible for *Helicobacter* species using the latest MALDI database (Berlamont et al., 2021). Indeed, there are currently only two *H. canis* among the 24 *Helicobacter* entries in the MALDI Biotyper reference database library (Bruker Daltonics). This incompleteness of the mass spectrometry database and the particular culture conditions inducing a degradation of the MALDI-TOF MS fingerprint may lead to an inadequate identification by MALDI-TOF MS (Welker, 2011; Murray, 2012). Sending the organism out to a reference laboratory should therefore be considered in case of a challenging identification.

**TABLE 1:** Previous and current microbiological investigations of *H. canis* isolated from blood cultures.

Author	Detection in blood bottle	Time to detection	Microscopic examination	Positive subcultures	Time to grow	Colonies aspect	Identification	AST		Treatment	Outcome
Lardinois et al. (Current report)	1 out of 4 (1 AE)	3 days	Gram negative and spiral-shaped rods	Columbia, chocolate, MacConkey and Karmali agar Best with Brucella Microaerophilic atmosphere 35°C	3–5 days	Thin layer with a cloud-like appearance in the medium	16S rRNA sequencing on subculture	*MICs by E-tests* (μ*g/ml*)*:		Ceftriaxone i.v. for 6 weeks.	Bacteriological and clinical cure
								AM = 0.064 A/C = 0.047 CRO = 0.075 MP < 0.002 MZ < 0.016 (S)	CM = 2 CI > 32 TC < 0.016 (S) RI < 0.002 (S)		
Mihevc et al., 2021	2 out of 4 (2AE)	5 days	Gram negative spiral rods	Columbia sheep agar Microaerophilic atmosphere 37°C Blood agar, Columbia agar 5% horse blood, Columbia agar 5% sheep blood, Brucella agar Microaerophilic atmosphere 37 and 42°C	2 days 3–4 days	Shiny translucent colonies of round or irregular shape	16S rRNA sequencing and MALDI-TOF MS on subculture	*MICs by E-tests (*μ*g/ml):*		Ceftriaxone i.v. and peroral doxycycline for 14 days	Bacteriological and clinical cure
								A/C = 0.016 (S) CTX = 0.125 (S) CRO = 0.25 (S)	GM = 0.064 MP = 0.25		
Gutiérrez-Arroyo et al., 2017	2 out of 2 (2 Peds)	6 days	Thin, curved Gram-negative bacilli	*H. pylori* selective supplement (Dent) blood base agar Microaerophilic atmosphere 37 and 42°C	7 days	Not reported	16S rRNA sequencing on subculture	Not reported		Cefotaxime i.v. and Metronidazole I.V.	Brain death
Shakir et al., 2017	4 out of 4 (4 AE)	5 days	Small, curved Gram-negative rods	Sheep blood agar Microaerophilic atmosphere 35 and 42°C	3 days	Small and tan	16S rRNA sequencing on subculture	Not performed		Doxycycline for 7 days then peroral Amoxicillin-clavulanate for 8 weeks	Bacteriological and clinical cure
van der Vusse et al., 2014	1 out of 6 (1 ANA)	3 days	Unusual, slightly curved, gram negative rods	Failed to grow Aerobic and anaerobic atmosphere	Not reported	Not reported	16S rRNA sequencing on blood bottle	Not performed		Cefuroxime for 3 days then Ciprofloxacin for 10 days	Bacteriological and clinical cure
Abidi et al., 2013	4 bottles out of 6 (4 AE)	4 days	Rod-like organisms after acridine orange stain Gram-negative bacilli	Chocolate blood agar Microaerophilic 37 and 42°C (optimal)	2–3 days	Thin and oily film	16S rRNA sequencing and MALDI-TOF MS on subculture	Not performed		Oral doxycycline for 6 weeks and ceftriaxone i.v. for 2 weeks	Bacteriological and clinical cure
Alon et al., 2010	1st episode: 2 out of 4 (2 AE) 2nd episode: 3 out of 4 (2 AE and 1 ANA)	2 days 3 days	Unusual, spiral Gram-negative rods	Failed to grow Aerobic and anaerobic atmosphere	Not reported	Not reported	16S rRNA sequencing on blood bottle.	Not performed		Cefuroxime for 3 days then peroral amoxicillin, omeprazole, and clarithromycin for 4 weeks	Bacteriological and clinical cure
Prag et al., 2007	1 pediatric bottle	5 days	Small, Gram-negative spiral rods, 2–3 mm long	5% sheep blood, chocolate and Brucella agar. Best with 5% horse blood agar plates with yeast extract Aerobic, anaerobic and microaerophilic atmosphere 37 and 42°C	4 days	Very small	16S rRNA sequencing on subculture	Cephalothin 30 μg (R) Acid nalidixic 30 μg (S)		Ampicillin and gentamicin i.v. then peroral mecillinam for 10 days	Clinical recurrence
Leemann et al., 2006	1 out of 4 (1 AE)	3 days	Spiral-shaped bacteria on acridine orange staining	Brucella agar supplemented with hemin, vitamin K1, cysteine, and 5% sheep blood Anaerobic and microaerophilic atmosphere 37°C	Not mentioned	Small and grayish	16S rRNA sequencing on subculture	*MICs by E-tests:*		Amoxicillin-clavulanate for 10 days then ceftriaxone i.v. for 2 weeks	Clinical recurrence then bacteriological and clinical cure
								Amoxicillin = 0.38 A/C = 0.094 (S) CRO = 0.75 (S) P/T = 1	IMI = 0.047 MZ = 0.064 CM = 0.094		
Gerrard et al., 2001	2 out of 10 (2 AE)	Not mentioned	Gram-negative spiral organism	Tryptic soy agar with 5% sheep blood and chocolate agar. Aerobic and anaerobic atmosphere 35°C	3–4 days	Not mentioned	16S rRNA sequencing on subculture	Not performed		Ampicillin i.v, Gentamicin i.v. and Ciprofloxacin then doxycycline and Metronidazole for 5 months	Clinical recurrence then bacteriological and clinical cure
**Trends**	**50% of aerobic blood bottles**	**4 days**	**Gram-negative spiral shaped rods**	**Enriched non-selective media** **Microaerophilic atmosphere 35°C–42°C**	**3–4 days**	**Thin layer of small colonies**	**16S rRNA sequencing**	**Determine MICs if possible**		**No guidelines**	**Favorable**

AE, aerobic; A/C, amoxicillin-clavulanate; AM, ampicillin; ANA, anaerobic; AST, antimicrobial susceptibility testing; CI, ciprofloxacin; CM, clindamycin; CTX, cefotaxime; CRO, ceftriaxone; GM, gentamicin; IMI, imipenem; I.V., intravenous; MALDI-TOF, matrix-assisted laser desorption ionization-time of flight mass spectrometry; MIC, minimal inhibitory concentration; MP, meropenem; MZ, metronidazole; P/T, piperacillin-tazobactam; R, resistant; rRNA, ribosomal ribonucleic acid; RI, rifampicin; S, susceptible; TC, tetracycline. *See Supplementary Table 1.

Molecular methods such as 16S rRNA gene sequencing were therefore more reliable for final identification (Shakir et al., 2017). Although the search for *H. canis* in a rich digestive flora could be particularly difficult, it may be relevant to identify the source. Passive filtration over Columbia agar supplemented with 5% sheep blood agar (CBA) or direct plating on CBA plus cefoperazone, amphotericin B, and teicoplanin has been suggested to isolate the bacteria among others (Ochoa et al., 2019). Considering these growing features, *H. canis* is a fastidious germ challenging routine microbiology laboratories. As a result, the detection rate in clinical laboratories is certainly underestimated, and clinicians probably miss bacteremia, hence the importance of sufficient blood volume to increase the detection sensitivity of such a pathogen. Some routine laboratories do not even culture *Helicobacter* species but may rely on histopathological diagnostic methods or reference laboratories culturing this germ and using molecular methods for identification. A good communication between clinicians and microbiologist is needed in order to optimize sample growth conditions and to choose the most adequate antimicrobial therapy.

As no guidelines were available for AST, we chose to interpret measured MICs with either *H. pylori* or PK-PD EUCAST breakpoints (The European Committee on Antimicrobial Susceptibility Testing, 2021). Based on EUCAST recommendations, MIC values were reported and their interpretation was rendered with caution. Many previous reports decided not to perform AST due to fastidious growth and lack of interpretation criteria. Only two cases reported MICs by *E*-test method and those were similar to ours except for clindamycin (0.094 and 12 μg/ml, respectively) (Leemann et al., 2006; Mihevc et al., 2021). Low MICs were observed as in our report for penicillins, amoxicillin-clavulanate, carbapenems, and metronidazole. One report obtained a high MIC for piperacillin-tazobactam (1 μg/mL) (Leemann et al., 2006). We obtained identical MIC for ceftriaxone (0.75 μg/ml). We furthermore observed low MICs for tetracycline and rifampicin as opposed to another case reporting a high MIC for ciprofloxacin (> 32 μg/ml). In 2019, although it was performed on a strain isolated from dog stool, a study reported MICs by agar dilution method. The major divergent result in comparison to AST realized on human strains concerned third-generation cephalosporins as MICs were high for cefsulodin and cefoperazone (128 μg/ml). They additionally observed full resistance for glycopeptides and trimethoprim (Ochoa et al., 2019). The germ is commonly found to be resistant to cephalothin with a MIC of 32 μg/ml except in one report (Shen et al., 2001). All studies also observed *in vitro* susceptibility of the germ for nalidixic acid (Stanley et al., 1993; Fox et al., 1996; Foley et al., 1999; Shen et al., 2001; Prag et al., 2007).

Relevant clinical features of published reports and their relative frequencies are summarized in Table 2. Its ability to cause bacteremia is highlighted in 83% of cases, mainly in patients with underlying conditions. Two reports that did not describe bacteremia mentioned the detection of *H. canis* in patients with digestive symptoms, one with gastroenteritis and the other with duodenal ulcerations attributed to Crohn’s disease (Burnens et al., 1993; Tankovic et al., 2011). Multiple clinical similarities between most cases were observed such as recurrent fever, immunocompromised status, or comorbidities and close contact with pets. On the other hand, digestive symptoms such as diarrhea were not systematically found, which could potentially explain extra-digestive origin of bacteremia such as the skin. Indeed, plausible skin entry points were described such as cellulitis or as in our case a superficial wound (Gerrard et al., 2001; Leemann et al., 2006; Shakir et al., 2017). The pathogen was not exclusively found in adults as three cases were observed in children including a bacteremia in a healthy 7-month-old baby (Burnens et al., 1993; Prag et al., 2007; Gutiérrez-Arroyo et al., 2017).

**TABLE 2:** Clinical features of previous and current *Helicobacter canis* reports.

Variables^a^	*H. canis* reports *N* = 12	% or Min–max
**Demographics**		
Adults	9	75
Female	6	50
Age (years)	46.5	0.2–78.0
Underlying conditions in adults (*N* = 9)	8	89
Immunosuppressors	5	56
ESRD	3	33
Splenectomized	2	22
Others^b^	5	56
**Clinical findings**		
Fever	7	58
Skin abnormalities	4	33
Digestive symptoms	4	33
**Laboratory findings**		
Bacteremia	10	83
WBC at hospitalization (×10^9^/L)	10.350	7.900–15.000
CRP at hospitalization (mg/dl)	7.7	0–9.4
Close contact with pets	10^c^	83
Dog	8	67
Cat	4	33
**Management and outcome**		
Cephalosporin based antibiotherapy	7	58
In-hospital time (days)	5	3–15
Fatal outcome	1^d^	8

*^a^Variables expressed as number of cases reported (%) or median (minimum–maximum observed rank).*

*^b^Chronic pancreatitis, lymphoma, sarcoidosis, Crohn’s disease, or rheumatoid arthritis.*

*^c^Two cases did not mention the presence or absence of a pet.*

*^d^One case reported H. canis bacteremia in a 2-month infant with cardiorespiratory arrest. CRP, C-reactive protein; ESRD, End-stage renal disease; WBC, white blood cells.*

Despite one case reporting the sudden death of a 2-month-old infant with associated *H. canis* bacteremia (Gutiérrez-Arroyo et al., 2017), a positive outcome has been reported in 9 out of 10 bacteremia reports despite the presence of underlying conditions in most cases. This could potentially be explained by the *in vitro* susceptibility of *H. canis* to several antibiotics, including penicillins. However, it has been suggested that *in vitro* susceptibility of the pathogen may not correlate with clinical response (Gerrard et al., 2001). Indeed, three authors reported patients that had experienced clinical recurrence after initial treatment with a combination of intravenous ampicillin and gentamicin (Gerrard et al., 2001; Prag et al., 2007) or oral amoxicillin-clavulanate (Leemann et al., 2006). Continued treatment by oral ciprofloxacin or mecillinam also did not prevent recurrence. Another patient received an oral combination of amoxicillin and clarithromycin based on *H. pylori* eradication therapy and was cured after 4 weeks (Alon et al., 2010), thereby suggesting that the addition of a macrolide results in a favorable outcome. Initial treatment with oral doxycycline (Abidi et al., 2013; Shakir et al., 2017), intravenous cefuroxime (van der Vusse et al., 2014), and intravenous ceftriaxone as administered in our case report also showed positive outcomes. Switching to intravenous ceftriaxone or a combination of oral doxycycline and metronidazole also provided a clinical cure after an initial treatment failure (Gerrard et al., 2001; Leemann et al., 2006).

## Conclusion

To date, clinical cases with *H. canis* are poorly described in the literature and prevalence is most likely underestimated. This is partly due to the difficulty of laboratories to detect, culture, and identify the microorganism given the need for molecular methods. We were nevertheless able to identify microbiological trends concerning the reported bacteremias and particularly the need to consider at least 7 days from blood sampling to identification and even more for obtaining AST. The lack of treatment guidelines and the paucity of data about *in vivo* and *in vitro H. canis* antimicrobial susceptibility lead clinicians to adopt treatment strategies not guided by susceptibility testing. Here we suggested ceftriaxone, doxycycline, or metronidazole as the best therapeutic choices once the pathogen has been identified. Moreover, this review also identified common clinical features, especially the frequent association of bacteremia in immunocompromised patients with recurrent fever and a close contact with pets, especially dogs. Direct fecal-oral zoonotic transmission prior to bacteremia is therefore potentially the most likely to occur. Finally, this report emphasizes the need for communication between clinicians and microbiologists in order to optimize culture and detection conditions and to adapt a patient’s antibiotic therapy as best as possible.

## Author Contributions

BL and AV analyzed, interpreted, and revised the microbiological data. LB analyzed, interpreted, and revised the clinical data. BL wrote, reviewed, and submitted the manuscript. All authors read and approved the final manuscript.

## Conflict of Interest

The authors declare that the research was conducted in the absence of any commercial or financial relationships that could be construed as a potential conflict of interest.

## Publisher’s Note

All claims expressed in this article are solely those of the authors and do not necessarily represent those of their affiliated organizations, or those of the publisher, the editors and the reviewers. Any product that may be evaluated in this article, or claim that may be made by its manufacturer, is not guaranteed or endorsed by the publisher.

## References

[B1] Abidi M. Z. Wilhelm M. P. Neff J. L. Hughes J. G. Cunningham S. A. Patel R. (2013). *Helicobacter canis* bacteremia in a patient with fever of unknown origin: fig 1. *J. Clin. Microbiol.* 51 1046–1048. 10.1128/JCM.02548-12 23284025 PMC3592094

[B2] Alon D. Paitan Y. Ben-Nissan Y. Chowers M. (2010). Persistent *Helicobacter canis* bacteremia in a patient with gastric lymphoma. *Infection* 38 62–64. 10.1007/s15010-009-9067-6 19756417

[B3] Berlamont H. De Witte C. De Bruyckere S. Fox J. G. Backert S. Smet A. (2021). Differentiation of gastric *Helicobacter* species using MALDI-TOF mass spectrometry. *Pathogens* 10:366. 10.3390/pathogens10030366 33803832 PMC8003121

[B4] Burnens A. P. Stanley J. Schaad U. B. Nicolet J. (1993). Novel *Campylobacter*-like organism resembling *Helicobacter fennelliae* isolated from a boy with gastroenteritis and from dogs. *J. Clin. Microbiol.* 31 1916–1917. 10.1128/JCM.31.7.1916-1917.1993 8349774 PMC265659

[B5] Ekman E. Fredriksson M. Trowald-Wigh G. (2013). *Helicobacter* spp. in the saliva, stomach, duodenum and faeces of colony dogs. *Vet. J.* 195 127–129. 10.1016/j.tvjl.2012.05.001 22683393

[B6] Foley J. E. Marks S. L. Munson L. Melli A. Dewhirst F. E. Yu S. (1999). Isolation of *Helicobacter canis* from a colony of Bengal cats with endemic diarrhea. *J. Clin. Microbiol.* 37 3271–3275. 10.1128/JCM.37.10.3271-3275.1999 10488191 PMC85546

[B7] Fox J. G. Drolet R. Higgins R. Messier S. Yan L. Coleman B. E. (1996). *Helicobacter canis* isolated from a dog liver with multifocal necrotizing hepatitis. *J. Clin. Microbiol.* 34 2479–2482. 10.1128/JCM.34.10.2479-2482.1996 8880504 PMC229299

[B8] Gerrard J. Alfredson D. Smith I. (2001). Recurrent bacteremia and multifocal lower limb cellulitis due to *Helicobacter* -like organisms in a patient with X-linked hypogammaglobulinemia. *Clin. Infect. Dis.* 33 e116–e118. 10.1086/323405 11595979

[B9] Gutiérrez-Arroyo A. Falces-Romero I. Corcuera-Pindado M. T. Romero-Gómez M. P. (2017). Bacteraemia in a two month-old infant. *Enferme. Infecc. Microbiol. Clin. Engl. Ed.* 35 676–677. 10.1016/j.eimce.2017.11.012 27435039

[B10] Leemann C. Gambillara E. Prod’hom G. Jaton K. Panizzon R. Bille J. (2006). First case of bacteremia and multifocal cellulitis due to *Helicobacter canis* in an immunocompetent patient. *J. Clin. Microbiol.* 44 4598–4600. 10.1128/JCM.01453-06 17005753 PMC1698384

[B11] Liu J. He L. Haesebrouck F. Gong Y. Flahou B. Cao Q. (2015). Prevalence of coinfection with gastric non- *Helicobacter pylori Helicobacter* (NHPH) species in *Helicobacter pylori* -infected patients suffering from gastric disease in Beijing, China. *Helicobacter* 20 284–290. 10.1111/hel.12201 25510739

[B12] Ménard A. Péré-Védrenne C. Haesebrouck F. Flahou B. (2014). Gastric and enterohepatic *Helicobacters* other than *Helicobacter pylori*. *Helicobacter* 19 59–67. 10.1111/hel.12162 25167947

[B13] Mihevc M. Koren Krajnc M. Bombek Ihan M. Holc I. (2021). *Helicobacter canis* bacteraemia in a rheumatoid arthritis patient treated with tofacitinib: case report and literature review. *Ann. Clin. Microbiol. Antimicrob.* 20:22. 10.1186/s12941-021-00426-x 33827581 PMC8028780

[B14] Momtaz H. Dabiri H. Souod N. Gholami M. (2014). Study of *Helicobacter pylori* genotype status in cows, sheep, goats and human beings. *BMC Gastroenterol.* 14:61. 10.1186/1471-230X-14-61 24708464 PMC4234145

[B15] Murray P. R. (2012). What is new in clinical microbiology—microbial identification by MALDI-TOF mass spectrometry. *J. Mol. Diagn.* 14 419–423. 10.1016/j.jmoldx.2012.03.007 22795961 PMC3427873

[B16] Ochoa S. Martínez O. A. Fernández H. Collado L. (2019). Comparison of media and growth conditions for culturing enterohepatic *Helicobacter* species. *Lett. Appl. Microbiol.* 69 190–197. 10.1111/lam.13192 31220348

[B17] Ochoa S. Ojeda J. Martínez O. A. Vidal-Veuthey B. Collado L. (2021b). Exploring the role of healthy dogs as hosts of enterohepatic *Helicobacter* species using cultivation-dependent and -independent approaches. *Zoonoses Public Health* 68 344–352. 10.1111/zph.12817 33586362

[B18] Ochoa S. Fernández F. Devotto L. France Iglesias A. Collado L. (2021a). Virulence assessment of enterohepatic *Helicobacter* species carried by dogs using the wax moth larvae *Galleria mellonella* as infection model. *Helicobacter* 26:e12808. 10.1111/hel.12808 33884706

[B19] Prag J. Blom J. Krogfelt K. A. (2007). *Helicobacter canis* bacteraemia in a 7-month-old child. *FEMS Immunol. Med. Microbiol.* 50 264–267. 10.1111/j.1574-695X.2007.00271.x 17567285

[B20] Sabry M. A. Abdel-Moein K. A. Seleem A. (2016). Evidence of zoonotic transmission of *Helicobacter canis* between sheep and human contacts. *Vector Borne Zoonotic Dis.* 16 650–653. 10.1089/vbz.2016.1994 27529744

[B21] Shakir S. M. Powers-Fletcher M. V. Slechta E. S. Fisher M. A. (2017). *Helicobacter* canis bacteraemia and cellulitis in a patient with end-stage renal disease. *JMM Case Rep.* 4:e005126. 10.1099/jmmcr.0.005126 29255610 PMC5729898

[B22] Shen Z. Feng Y. Dewhirst F. E. Fox J. G. (2001). Coinfection of enteric *Helicobacter* spp. and *Campylobacter* spp. in cats. *J. Clin. Microbiol.* 39 2166–2172. 10.1128/JCM.39.6.2166-2172.2001 11376052 PMC88106

[B23] Stanley J. Linton D. Burnens A. P. Dewhirst F. E. Owen R. J. Porter A. (1993). *Helicobacter canis* sp. nov., a new species from dogs: an integrated study of phenotype and genotype. *J. Gen. Microbiol.* 139 2495–2504. 10.1099/00221287-139-10-2495 8254320

[B24] Swennes A. G. Turk M. L. Trowel E. M. Cullin C. Shen Z. Pang J. (2014). *Helicobacter canis* colonization in sheep: a zoonotic link. *Helicobacter* 19 65–68. 10.1111/hel.12097 24188726 PMC3897236

[B25] Tabrizi S. Atashi A. (2015). Identification of *Helicobacter* spp. in gastrointestinal tract, pancreas and hepatobiliary system of stray cats. *Iran. J. Vet. Res.* 16 374–376. 27175206 PMC4782678

[B26] Tankovic J. Smati M. Lamarque D. Delchier J.-C. (2011). First detection of *Helicobacter canis* in chronic duodenal ulcerations from a patient with Crohn’s disease. *Inflamm. Bowel Dis.* 17 1830–1831. 10.1002/ibd.21610 21744440

[B27] The European Committee on Antimicrobial Susceptibility Testing (2021). *Breakpoint Tables for Interpretation of MICs and Zone Diameters, Version 11.0.* Available online at: http://www.eucast.org (accessed November 13, 2021).

[B28] van der Vusse M. L. van Son W. J. Ott A. Manson W. (2014). *Helicobacter canis* bacteremia in a renal transplant patient. *Transpl. Infect. Dis.* 16 125–129. 10.1111/tid.12174 24372779

[B29] Wauters G. Avesani V. Laffineur K. Charlier J. Janssens M. Van Bosterhaut B. (2003). *Brevibacterium lutescens* sp. nov., from human and environmental samples. *Int. J. Syst. Evol. Microbiol.* 53 1321–1325. 10.1099/ijs.0.02513-0 13130013

[B30] Welker M. (2011). Proteomics for routine identification of microorganisms. *Proteomics* 11 3143–3153. 10.1002/pmic.201100049 21726051

