# Supplementary figures and images for: Helicobacter canis: A Review of Microbiological and Clinical Features
Source: Front Microbiol. 2022 Feb 23;12:814944. doi: 10.3389/fmicb.2021.814944 (PMC8905544; doi:10.3389/fmicb.2021.814944)

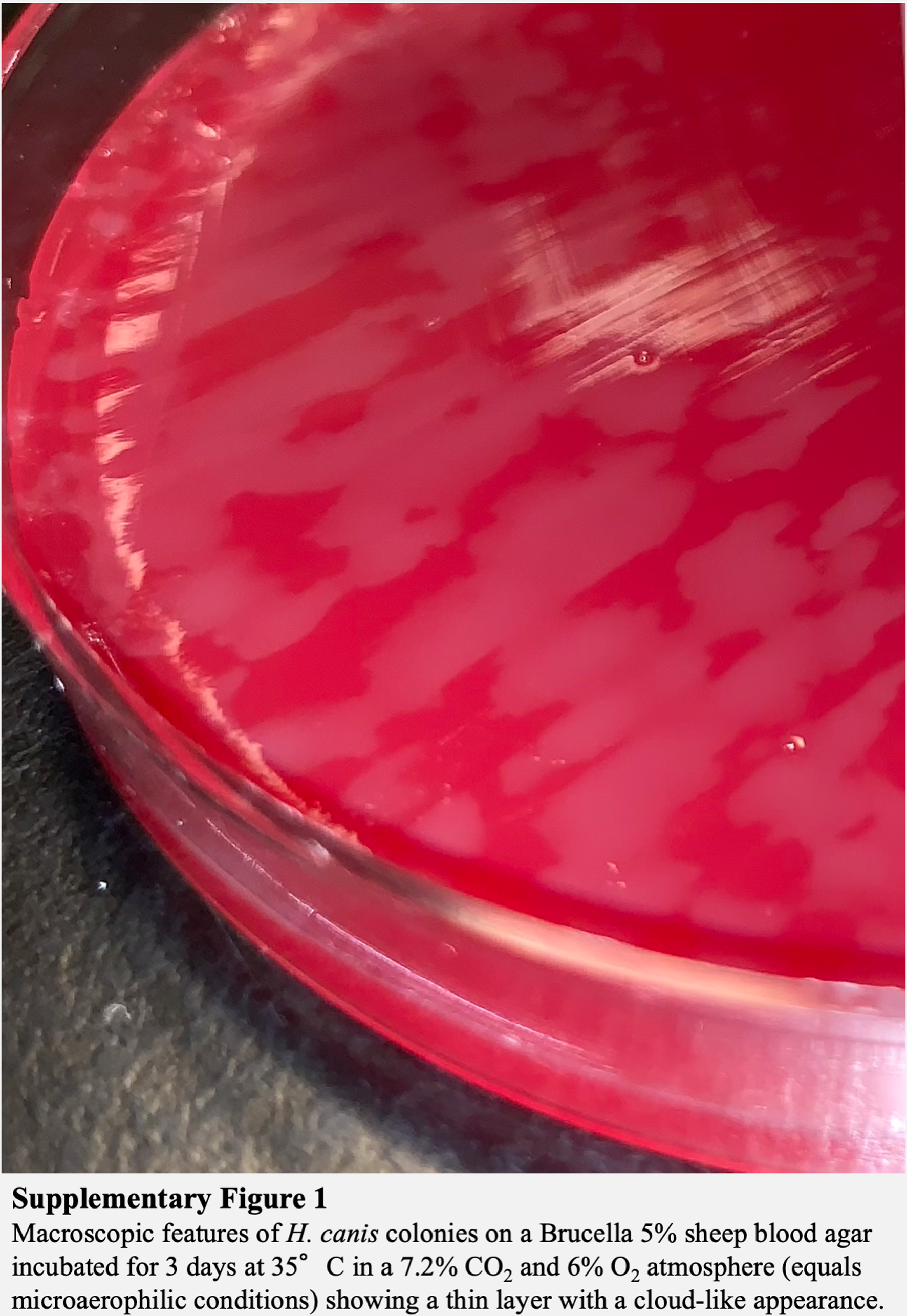

Supplement: Supplementary file 1 [file Image_1.tiff]

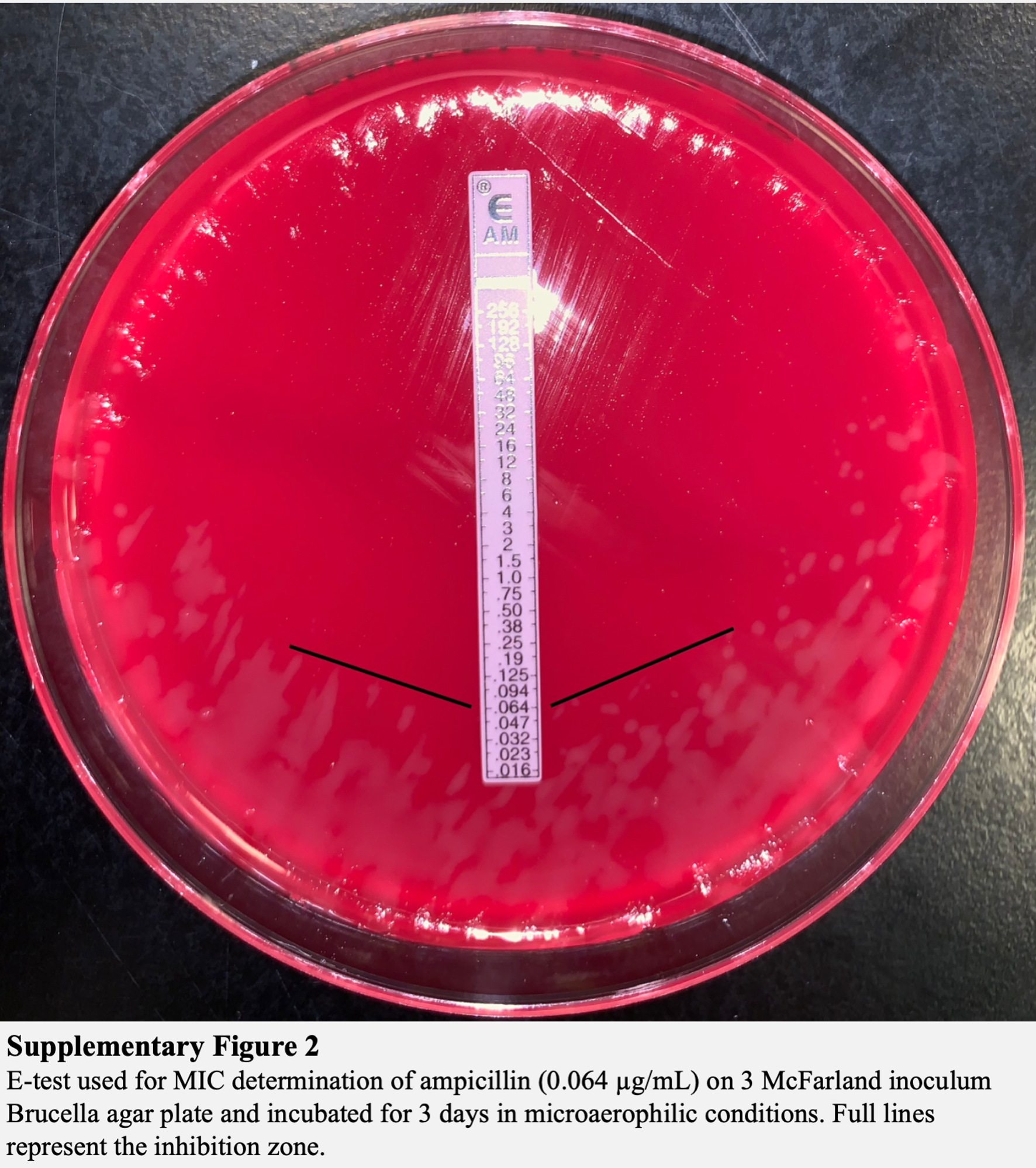

Supplement: Supplementary file 2 [file Image_2.tiff]
