# Supplementary material for: Helicobacter canis: A Review of Microbiological and Clinical Features
Source: Front Microbiol. 2022 Feb 23;12:814944. doi: 10.3389/fmicb.2021.814944 (PMC8905544; doi:10.3389/fmicb.2021.814944)
Supplement: Supplementary file 3 [file Data_Sheet_1.docx]

| **Supplementary table**Antimicrobial susceptibiltytestings of *H. Canis* current report | | | |
| --- | --- | --- | --- |
| E-test INN(Concentration in µg/mL) | MIC (µg/mL) | Interpretation | Breakpoints |
| Rifampicine RI (0.002-32) | <0.002 | Susceptible | *H. pylori* EUCAST 2021 |
| Métronidazole MZ (0.016-256) | <0.016 | Susceptible | *H. pylori* EUCAST 2021 |
| Tétracycline TC (0.016-256) | < 0.016 | Susceptible | *H. pylori* EUCAST 2021 |
| Ceftriaxone TX (0.002-32) | = 0.75 | To use with caution | PK-PD EUCAST 2021 |
| Amoxi-clav XL (0.016-256) | = 0.047 | To use with caution | PK-PD EUCAST 2021 |
| Ampicilline AM (0.016-256) | = 0.064 | To use with caution | PK-PD EUCAST 2021 |
| Méropénem MP (0.002-32) | < 0.002 | To use with caution | PK-PD EUCAST 2021 |
| CiprofloxacineCI (0.002-32) | > 32 | Should not be used | PK-PD EUCAST 2021 |
| Clindamycine CM (0.016-256) | = 12 | Uninterpretable | No breakpoint nor wild type distribution MIC |
| INN, International nonproprietary name; MIC, Minimal inhibitory concentration | | | |
